# The Neuroprotective Effects of Cinnamic Aldehyde in an MPTP Mouse Model of Parkinson’s Disease

**DOI:** 10.3390/ijms19020551

**Published:** 2018-02-12

**Authors:** Woom-Yee Bae, Jae-Sun Choi, Joo-Won Jeong

**Affiliations:** 1 Department of Biomedical Science, Graduate School, Kyung Hee University, Seoul 02447, Korea; woom8875@khu.ac.kr; 2 Department of Anatomy and Neurobiology, College of Medicine, Kyung Hee University, Seoul 02447, Korea; ChoiJS@khu.ac.kr

**Keywords:** cinnamic aldehyde, autophagy, Parkinson’s disease, MPTP, MPP^+^

## Abstract

Cinnamic aldehyde (CA), a key flavor compound in cinnamon essential oil, has been identified as an anti-oxidant, anti-angiogenic, and anti-inflammatory material. Recently, the neuroprotective effects of CA have been reported in various neurodegenerative disorders, including Parkinson’s disease (PD). In neurons, autophagy is tightly regulated, and consequently, the dysregulation of autophagy may induce neurodegenerative disorders. In the present study, we found that the selective dopaminergic neuronal death in the substantia nigra of 1-methyl-4-phenyl-1,2,3,6-tetrahydropyridine (MPTP) mouse models was prevented by CA. Stimulation of microtubule-associated protein light chain 3 (LC3) puncta mediated by MPTP treatment was decreased by CA. Moreover, down-regulated p62 in the substantia nigra of MPTP mice was increased by administration of CA. Finally, we showed that blockage of autophagy using autophagy inhibitors protected the 1-methyl-4-phenylpyridinium (MPP^+^)-mediated death of BE(2)-M17 cells. Together these results suggest that CA has a neuroprotective effect in a PD model and that inhibition of autophagy might be a promising therapeutic target for PD.

## 1. Introduction

Parkinson’s disease (PD) is a progressive neurodegenerative disorder and is characterized by selective dopaminergic neuronal loss in the substantia nigra and striatum of the brain, which leads to accountable motor symptoms, including bradykinesia, resting tremor, rigidity, and postural instability [1]. To date, researchers have attempted to find effective medicine for PD, and current therapies or drugs aim at stopping or slowing the progression of PD [2]. The main pathological pathway responsible for the dopaminergic neuronal death in PD is still unidentified [3]. Mounting evidence suggests that PD might be related to dysregulation of autophagy in neuronal cells, astrocytes, and microglial cells [4,5,6]. Therefore, recent studies on PD interventions have focused on the regulation and function of autophagy in PD.

1-Methyl-4-phenyl-1,2,3,6-tetrahydropyridine (MPTP) is a neurotoxin that has the ability to cross the blood–brain barrier and damage dopaminergic neurons in the nigrostriatal pathway [7]. MPTP is transformed by the enzyme monoamine peroxidase into 1-methyl-4-phenylpyridinium (MPP^+^), which binds to dopamine transporters, causing the inhibition of dopamine uptake and depletion of its cerebral levels [8]. Because MPTP induces neurotoxicity in the nigrostriatal pathway and MPP^+^ stimulates neuronal cell death, MPTP and MPP^+^ are used to initiate PD in animal models and cellular models, respectively [9,10,11,12].

**Figure 1:**
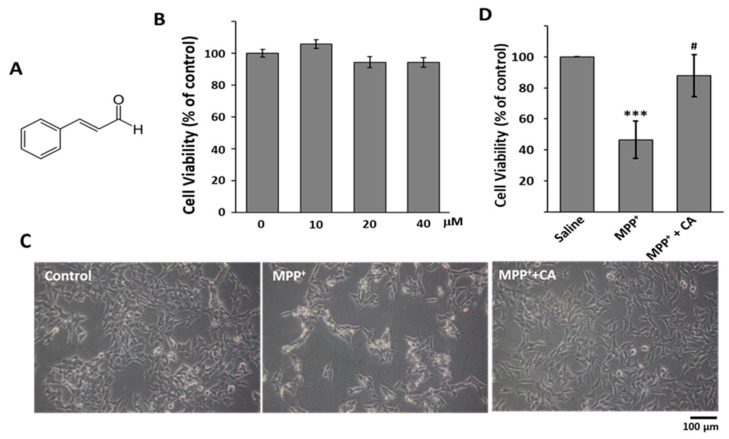
The effects of CA on MPP^+^-induced cell death. (**A**) The chemical structure of CA; (**B**) BE(2)-M17 cells were incubated with the indicated concentration of CA for 48 h. The data are expressed as the means ± standard deviation (S.D.) of three independent experiments; (**C**) 0.5 mM MPP^+^ was added to cells with 10 μM of CA for 48 h. Photos of cells were taken as indicated; (**D**) The live cell number was counted, and the data are expressed as the means ± S.D. of three independent experiments. *** *p* < 0.001 versus control; ^#^ *p* < 0.05 versus MPP^+^-treated cells.

Because PD is the progressive disorder, investigators are searching for neuroprotective agents that are capable of stopping the underlying pathological condition and preventing further neuronal death. Most clinical drugs having neuroprotective effects are synthetic drugs that show undesirable adverse effects or toxicity [13,14]. Because natural products are considered to be relatively safe and effective, many researchers have tried to find their associated neuroprotective effects. Cinnamic aldehyde (cinnamaldehyde, CA) is a natural product extracted from *Cinnamon* trees [15,16]. As a yellow and viscous liquid, CA (Figure 1A) constitutes 98% of the essential oil of Cinnamon bark. Recently, CA was reported to protect individuals from neurodegenerative disorders, such as Alzheimer’s disease and PD [17,18]. However, whether CA shows anti-PD activity or affects autophagy regulation is still unknown.

In the present study, the MPTP mouse model and the MPP^+^-induced cell injury model were used to examine the neuroprotective effects of CA in vivo and in vitro. We demonstrated that CA significantly reduced the selective dopaminergic cell death in the substantia nigra and striatum of the MPTP-administered mice. CA reduced LC3 puncta stimulated by MPTP and MPP^+^ treatment, whereas CA increased p62 that had been reduced by MPTP and MPP^+^ treatment. These findings suggest that CA could block dysregulated autophagy under PD conditions. In addition, inhibition of autophagy also reduced MPP^+^-mediated cell death. Collectively, our results indicated that CA is a novel natural product that regulates autophagy in cell death under PD conditions and that CA may be a promising agent for the treatment of PD.

## 2. Results

### 2.1. CA Recovered MPP^+^-Induced Cell Death in BE(2)-M17 Cells

To evaluate the effects of CA on MPP^+^-induced neuronal cell death, we used BE(2)-M17 cells, a human neuroblastoma cell line. Cells were treated with various concentrations of CA for 48 h, and the viability was examined by MTT assay. Because CA did not show any significant effects on cell viability up to 40 μM (Figure 1B), we selected 10 μM of CA for further experiments in this study. When the cells were exposed to 0.5 mM of MPP^+^ for 48 h, cell viability was reduced by almost 50%. The reduction of viability mediated by MPP^+^ was significantly recovered by CA treatment (Figure 1C,D), indicating that CA attenuates MPP^+^-induced cell death in a cellular PD model.

### 2.2. CA Protected against MPTP-Induced Dopaminergic Cell Death

**Figure 2:**
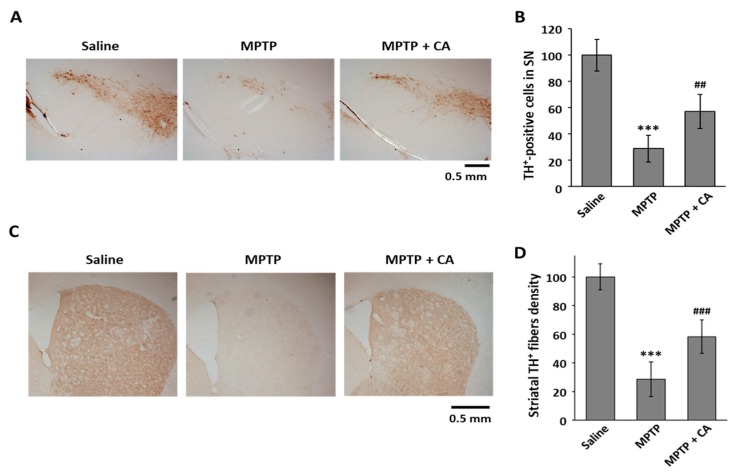
The effects of CA on dopaminergic cell death in an MPTP mouse model. (**A**) TH was stained using coronal substantia nigra sections from each group as indicated; (**B**) TH^+^-cell bodies were counted, and the data are expressed as the means ± S.D. of three independent experiments. *** *p* < 0.001 versus saline group; ^##^ *p* < 0.01 versus the MPTP group; (**C**) Striatum sections of each group as indicated were stained with anti-TH antibody; (**D**) TH^+^ fibers were quantified using ImageJ software. Quantitative values were normalized to 100% of saline mice. *** *p* < 0.001 versus saline group; ^###^ *p* < 0.001 versus MPTP group.

To clarify the neuroprotective effect of CA in PD, we used an MPTP mouse model. CA (10 mg/kg, i.p.) was administered daily for a week to MPTP-intoxicated mice. To examine whether CA is toxic in this animal model, we measured the hepatic toxicity marker enzymes, including aspartate aminotransferase (AST) and alanine aminotransaminase (ALT). As shown in Table 1, 10 mg/kg of CA did not show any cytotoxicity in the MPTP-treated mice. To identify the dopaminergic neuronal cell death in a mouse PD model, we stained the tissues of the substantia nigra and striatum using a specific antibody against tyrosine hydroxylase (TH), which is a marker for dopaminergic neurons. The administration of CA prevented the selective loss of TH-positive cell death mediated by MPTP injection in the substantia nigra (Figure 2A,B). Consistent with dopamine neuronal protection in the substantia nigra, CA prevented the severe loss of dopamine neuron fiber density from MPTP intoxication in the striatum (Figure 2C,D).

### 2.3. CA Inhibits Autophagy Stimulated in the Substantia Nigra of MPTP-Treated Mice

**Figure 3:**
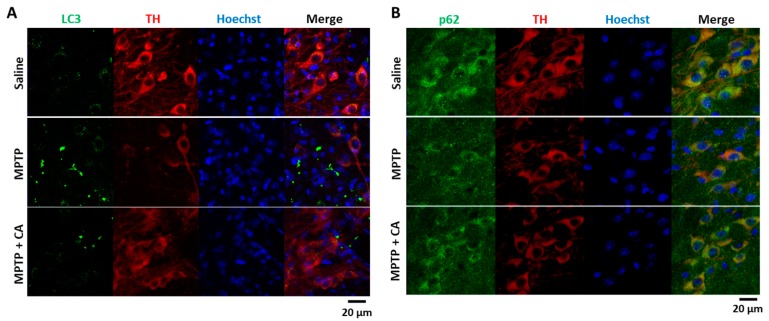
The effects of CA on autophagy in the substantia nigra of MPTP-treated mice. (**A**,**B**) Immunofluorescent staining was performed using specific antibodies. The substantia nigra tissues from each group were stained with anti-LC3 antibody (green, **A**), anti-p62 (green, **B**), anti-TH antibody (red), and Hoechst33342 (blue).

It is believed that autophagy is an important event in controlling cell death. Moreover, many recent studies have reported that autophagy is induced in MPTP-treated animals [19,20]. Therefore, we wanted to determine whether CA could regulate autophagy in a PD model. As shown in Figure 3A, LC3 puncta were stimulated in the substantia nigra of MPTP-treated mice. Conversely, treatment with CA decreased MPTP-induced LC3 puncta (Figure 3A). However, it was unclear whether the increase in LC3 puncta was associated with autophagy flux; therefore, we also examined the distribution of p62. The cytoplasmic expression level of p62 was decreased in MPTP-treated mice, and CA recovered the reduction of p62 expression (Figure 3B). From these results, we suggest that CA reduced autophagy stimulated in the MPTP mouse model. 

### 2.4. CA Decreased Autophagy in MPP^+^-Treated BE(2)-M17 Cells

**Figure 4:**
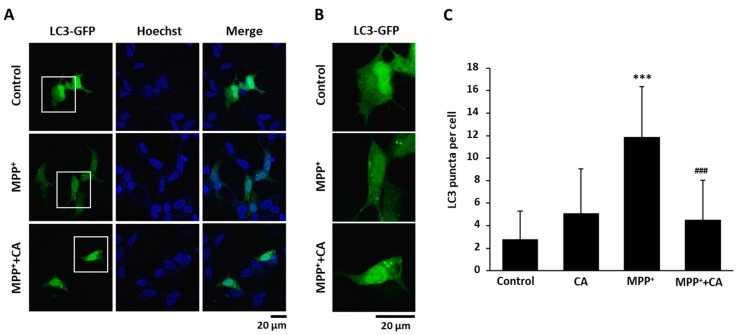
The effects of CA on LC3 puncta formation in an MPP^+^ cellular PD model. (**A**) BE(2)-M17 cells were transfected with a GFP-tagged LC3 expression vector and then with either DMSO or 10 μM of CA in the presence of 0.5 mM of MPP^+^ for 48 h. LC3 puncta were visualized as a GFP signal. Cell nuclei (blue) were stained with Hoechst33342; (**B**) The magnified versions of white squares in (**A**); (**C**) The puncta with GFP signals in a cell were counted and graphed. *** *p* < 0.001 versus control; ^###^ *p* < 0.001 versus MPP^+^-treated cells.

Next, to explore whether CA regulates autophagy under PD conditions, we treated BE(2)-M17 cells with MPP^+^. After the LC3-GFP expression vector was transfected, LC3 puncta were examined by fluorescence microscopy and counted. As shown in Figure 4A,B, MPP^+^ treatment stimulated the formation of LC3 puncta, and conversely, treatment with CA significantly decreased the puncta.

**Figure 5:**
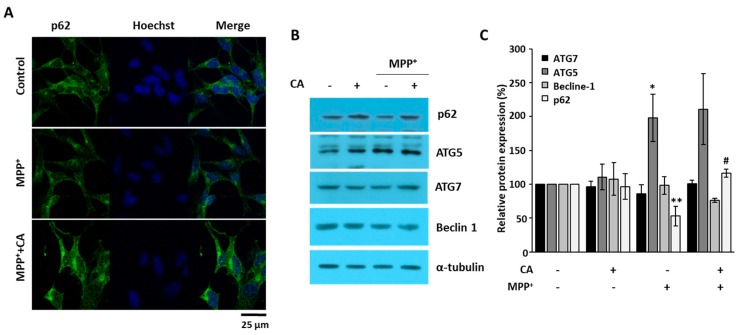
The effects of CA on autophagy-related protein expression. (**A**) BE(2)-M17 cells were treated with either vehicle or 10 μM of CA in the presence of 0.5 mM of MPP^+^ for 48 h. Intracellular expression of p62 (green) was determined by immunofluorescent staining using a specific antibody. Cell nuclei (blue) were stained with Hoechst33342; (**B**) Western blots from whole cell lysates were performed using anti-p62, -beclin 1, -ATG5, -ATG7, and anti-α-tubulin antibodies; (**C**) Relative expression was quantified and plotted. * *p* < 0.05, ** *p* < 0.01 versus control; ^#^ *p* < 0.05 versus MPP^+^-treated cells.

When p62 protein expression was examined using immunofluorescence, we found that p62 was decreased in MPP^+^-treated cells. Moreover, CA recovered the reduction of p62 in MPP^+^-treated cells (Figure 5A). To examine the expression of autophagy-related proteins, we performed Western blotting analysis. As shown in Figure 5B, beclin 1, ATG5, and ATG7 were not changed by CA in the presence of MPP^+^. However, p62 was decreased by MPP^+^ and recovered by CA (Figure 5B), suggesting that CA inhibits autophagy in MPP^+^-induced cell death.

### 2.5. Inhibition of Autophagy Suppressed MPP^+^-Induced Cell Death

**Figure 6:**
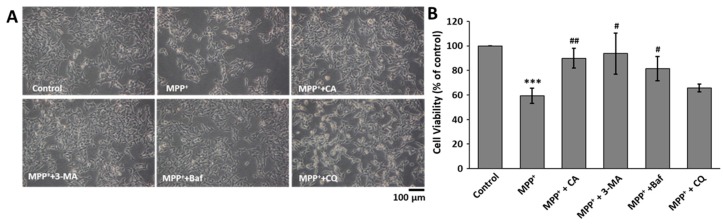
The effects of autophagy inhibitors on MPP^+^-induced cell death. (**A**) BE(2)-M17 cells were treated with 10 nM of bafilomycin A1, 1 mM of 3-MA, 10 μM chloroquine, and 10 μM of CA in the presence of 0.5 mM of MPP^+^ for 48 h. Photos were taken of the cell morphologies under each condition. Baf: bafilomycin A1; CQ: chloroquine; (**B**) The live cell number was counted, and the data are expressed as the means ± S.D. of three independent experiments. *** *p* < 0.001 versus control; ^#^ *p* < 0.05, ^##^ *p* < 0.01 versus MPP^+^-treated cells.

We already found that CA decreased MPP^+^-mediated cell death and inhibited autophagy stimulated by MPP^+^. Therefore, we wanted to assess whether inhibition of autophagy affected MPP^+^-mediated cell death. In the same manner as the treatment with CA, bafilomycin A1 and 3-MA significantly recovered MPP^+^-mediated BE(2)-M17 cell death (Figure 6A,B). However, chloroquine could not recover the cell death. Collectively, we suggested that CA suppressed MPP^+^-induced cell death by blocking autophagy.

## 3. Discussion

Accumulating studies indicated that misfolding of α-synuclein, imbalanced autophagy, and mitochondrial dysfunction are deeply involved in the pathogenesis of PD [21]. Therapies such as oral L-dopa and deep brain stimulation are used in the clinic to improve motor complications of PD [22]. However, these therapies are not ideal to stop or slow down PD and even show side effects such as dyskinesia and morning akinesia. Natural products are considered relatively safe with limited side effects and may become potential drugs for PD treatment. Here, we selected CA in our study to understand its influence on MPTP- and MPP^+^-induced PD models. We, for the first time, found that CA significantly rescued MPP^+^-induced cell death and MPTP-induced dopaminergic cell death (Figure 1 and Figure 2).

The major physiological role of autophagy is recycling intracellular energy resources in response to conditions of nutrient depletion [23]. Another function is degradation of cytotoxic proteins and damaged organelles under stress conditions [24]. The pathogenesis of numerous neurodegenerative diseases has been linked cytoplasmic and nuclear inclusions composed of aggregated or polyubiquitinated proteins. These include amyloid plaques of Alzheimer’s disease and the Lewy bodies of PD. The protein aggregations in these diseases are involved in synaptic dysfunction and neuronal degeneration. According to the recent reports, autophagy-related mechanisms lead to pathological alterations of neurodegeneration [25,26]. In this study, we wanted to know whether CA could regulate autophagy under PD conditions. In MPTP-administered mice and MPP^+^-injured cells, LC3 puncta were strongly increased, and the protein level of p62 was decreased (Figure 3, Figure 4 and Figure 5). These autophagic changes were recovered with the administration of CA, indicating that CA rehabilitates dysregulated autophagy under PD conditions.

Recently, many reports have shown that autophagy is induced under PD conditions, including with MPP^+^ treatment and in MPTP mice [27,28]. However, the function of stimulated autophagy is not clear. Neurons require a basal level of autophagic degradation to mediate replacement of damaged components or to facilitate synaptic remodeling. Under PD conditions, the neuronal loss in the substantia nigra is partly due to the accumulation of aggregated and/or misfolded proteins [21]. Most aggregated and/or misfolded proteins are cleaned via two important pathways: the ubiquitin proteasome system and the autophagy-lysosome pathway [29]. Because misfolded α-synuclein can be cleared by the autophagic pathway [30], it is easily predicted that autophagy will induce cell survival in PD. However, in the present study, we found that autophagy inhibitors such as 3-MA and bafilomycin A1 rescued MPP^+^-induced cell death in BE(2)-M17 cells (Figure 6). In addition, at the identical concentrations we used in this study, these autophagy inhibitors had no significant effect on basal BE(2)-M17 cell viability. These results were consistent with the recent report that neither 3-MA nor wortmannin were able to inhibit the increase in autophagic vacuoles induced by MPP^+^ [31]. Moreover, a recent report suggested that stimulated autophagy induces cell death in MPP^+^-treated human neuroblastoma cells [32]. In many diseases, autophagy is stimulated by various stress conditions, and autophagic cell death is induced [33,34,35]. However, many reports were inconsistent with our data [36,37]. This inconsistency may be attributed to the analysis of different cell types and conditions among the studies. In addition, autophagy can induce either cell survival or cell death depending on the environment [38], and autophagic stress often induces impaired degradation of misfolded proteins [39]. The mechanisms and functions of autophagic perturbation are not mutually exclusive.

In the present study, MPTP and MPP^+^ were able to induce autophagy in neuronal cells, which was shown to exert cellular death under PD conditions. In addition, CA was able to protect dopaminergic neuronal cells from the toxic effects of MPP^+^ by reducing the rate of autophagy, thus supporting the hypothesis that autophagy induced by MPP^+^ treatment may stimulate cell death. The results of the present study may help to elucidate the important role of autophagy under PD conditions and to direct the development of a novel strategy to treat patients with PD. Moreover, we strongly support the future use of CA for the treatment of PD and related complications.

## 4. Materials and Methods

### 4.1. Materials

MPP^+^, MPTP, CA, bafilomycin A1, 3-methyladenine (3-MA), and chloroquine were purchased from Sigma Aldrich (St Louis, MO, USA). Antibodies for tyrosine hydroxylase (TH) was purchased from Cell Signaling Technology (Beverly, MA, USA). Antibodies recognizing p62, beclin 1, ATG5, and ATG7 were obtained from Santa Cruz Biotechnology (Dallas, TX, USA). A mammalian expression vector for LC3-GFP was kindly provided by Joohun Ha (Kyung Hee University, Seoul, Korea).

### 4.2. Cell Culture, Transfection, and Treatment

Human neuroblastoma BE(2)-M17 cells were maintained in Dulbecco’s modified Eagle’s medium (WelGENE, Korea) supplemented with 10% fetal bovine serum (HyClone, Logan, UT, USA) and antibiotics at 37 °C in a humidified atmosphere of 95% air and 5% CO_2_. MPP^+^ and CA were added to the cells for 48 h. Transient transfection of the LC3-GFP expression vector was conducted using the polyethylenimine method. The cells were also treated with bafilomycin A1, 3-MA, or chloroquine in the presence of MPP^+^.

### 4.3. 3-(4,5-Dimethylthiazol-2-yl)-2,5-Diphenyltetrazolium Bromide (MTT) Assay

BE(2)-M17 cells were treated with MPP^+^ for 48 h, and MTT (0.1 mg/mL; Sigma Aldrich) was added to each well and incubated at 37 °C for 2 h. The media were removed, and DMSO was added to each well. The absorbance was measured at 560 nm using an iMark Microplate Absorbance Reader (Bio-Rad, Hercules, CA, USA).

### 4.4. Animals and MPTP Mouse Model

Ten-week-old male C57BL/6 mice (*n* = 4–5 per group) were purchased from Daehan Bio-link (Chungbuk, Korea). All experiments were performed in accordance with the approved animal protocols and guidelines established by Kyung Hee University (KHUASP(SE)-17-018, 26-04-2017). For MPTP intoxication, the mice received four intraperitoneal injections (i.p.) of MPTP (20 mg/kg) at 2 h intervals, as previously reported [40,41]. Injections of CA (10 mg/kg, i.p.) were delivered daily for 7 days after MPTP injection.

### 4.5. Immunohistochemistry and Immunofluorescent Analyses

Animals were transcardially perfused with 4% paraformaldehyde. Brains were then isolated, frozen, and cut into 30 μm slices on a freezing microtome (Leica Microsystems, Bensheim, Germany). The sections (*n* = 3–4 per mouse) were then incubated with PBS containing 0.3% Triton X-100 for 20 min and stained with specific antibodies against TH, LC3, and p62. The antigen sites were visualized either with the VECTASTAIN Elite ABC Kit (VECTOR laboratories, Burlingame, CA, USA) and 3,3-diaminobenzidine (Sigma-Aldrich, St Louis, MO, USA) or with Alexa-488 (Invitrogen, Eugene, OR, USA) and Alexa-594 (Invitrogen). The intensity of each staining was measured using ImageJ software 1.51n (Wayne Rasbang, NIH, Bethesda, MD, USA).

### 4.6. Western Blotting Analysis

Total proteins were isolated from the indicated cells using the lysis buffer (10 mM Tris, 10 mM NaCl and 0.2% NP-40) containing a protease inhibitor cocktail (Sigma Aldrich) and phosphatase inhibitors (1 mM Na_3_VO_4_ and 10 mM NaF). The equal amounts of total proteins were separated by SDS-PAGE, and were transferred onto a PVDF membrane (Millipore Corporation, Billerica, MA, USA). The membranes were blocked with 5% bovine serum albumin (BSA) in Tris-buffered saline containing 0.1% tween 20 (TBS-T) and incubated overnight at 4 °C with primary antibodies diluted in blocking buffer. The membranes were washed and incubated with the appropriate HRP-conjugated secondary antibodies for 1 h at room temperature. The signal was detected using an ECL Western detection system (Thermo Scientific, Rockford, IL, USA).

### 4.7. Statistical Analysis

All experiments were performed at least three times. The results were expressed as the means ± S.D. Differences between groups were examined for statistical significance using a *t*-test. *p*-values <0.05 were considered statistically significant.

## Figures and Tables

**Table 1:** The effects of CA treatment on the activities of aspartate aminotransferase (AST) and alanine aminotransaminase (ALT).

Group	AST (IU/L)	ALT (IU/L)
Saline	26.9 ± 6.0	17.4 ± 9.0
Saline + CA	24.9 ± 4.7	12.2 ± 0.9
MPTP	28.6 ± 2.7	16.0 ± 0.6
MPTP + CA	30.8 ± 6.4	15.7 ± 1.2

Values are means ± S.D. for three mice in each group.

## Abbreviations

CA	Cinnamic Aldehyde
PD	Parkinson’s Disease
MPTP	1-methyl-4-phenyl-1,2,3,6-tetrahydropyridine
MPP^+^	1-methyl-4-phenylpyridinium
TH	tyrosine hydroxylase
